# *Schistosoma mansoni*-Mediated Suppression of Allergic Airway Inflammation Requires Patency and Foxp3^+^ Treg Cells

**DOI:** 10.1371/journal.pntd.0002379

**Published:** 2013-08-15

**Authors:** Laura E. Layland, Kathrin Straubinger, Manuel Ritter, Eva Loffredo-Verde, Holger Garn, Tim Sparwasser, Clarissa Prazeres da Costa

**Affiliations:** 1 Institute of Medical Microbiology, Immunology and Hygiene (MIH), Technische Universität München, Munich, Germany; 2 Institute of Medical Microbiology, Immunology and Parasitology (IMMIP), University Clinic Bonn, Bonn, Germany; 3 Institute of Laboratory Medicine and Pathobiochemistry, Medical Faculty, Philipps-University Marburg, Marburg, Germany; 4 Institut für Infektionsimmunologie TWINCORE - Zentrum für Experimentelle und Klinische Infektionsforschung GmbH, Hannover, Germany; Uniformed Services University of the Health Sciences, United States of America

## Abstract

The continual rise of asthma in industrialised countries stands in strong contrast to the situation in developing lands. According to the modified Hygiene Hypothesis, helminths play a major role in suppressing bystander immune responses to allergens, and both epidemiological and experimental studies suggest that the tropical parasitic trematode *Schistosoma mansoni* elicits such effects. The focus of this study was to investigate which developmental stages of schistosome infection confer suppression of allergic airway inflammation (AAI) using ovalbumin (OVA) as a model allergen. Moreover, we assessed the functional role and localization of infection-induced CD4^+^Foxp3^+^ regulatory T cells (Treg) in mediating such suppressive effects. Therefore, AAI was elicited using OVA/adjuvant sensitizations with subsequent OVA aerosolic challenge and was induced during various stages of infection, as well as after successful anti-helminthic treatment with praziquantel. The role of Treg was determined by specifically depleting Treg in a genetically modified mouse model (DEREG) during schistosome infection. Alterations in AAI were determined by cell infiltration levels into the bronchial system, OVA-specific IgE and Th2 type responses, airway hyper-sensitivity and lung pathology. Our results demonstrate that schistosome infection leads to a suppression of OVA-induced AAI when mice are challenged during the patent phase of infection: production of eggs by fecund female worms. Moreover, this ameliorating effect does not persist after anti-helminthic treatment, and depletion of Treg reverts suppression, resulting in aggravated AAI responses. This is most likely due to a delayed reconstitution of Treg in infected-depleted animals which have strong ongoing immune responses. In summary, we conclude that schistosome-mediated suppression of AAI requires the presence of viable eggs and infection-driven Treg cells. These data provide evidence that helminth derived products could be incorporated into treatment strategies that specifically target suppression of immune responses in AAI by inducing Treg cells.

## Author Summary

Infections with schistosomes, such as *S. mansoni*, *S. japonicum* and *S. haematobium*, are considered a major public health concern. Morbidity arises through granulomatous responses to eggs that become trapped in infected tissues. Interestingly, schistosomes belong to the group of helminths that have been shown to reduce allergy or autoimmunity. Indeed, the evidence provided by epidemiological surveys and experimental animal models has been so overwhelming that such helminths are now included in the Hygiene Hypothesis. However, since helminths provoke immunological responses that are similar to those seen in allergy (increased eosinophilia and IgE) it is suggested that additional mechanisms dampen such allergic responses. Helminth-induced regulatory T cells (Treg) are considered a component of these modulatory networks. Using an allergic airway inflammation model, we have elucidated that schistosome-mediated protection requires patency, that is, active egg production from fecund female worms. In addition, protection was shown to be mediated by infection-induced Treg. Interestingly, in endemic countries it is usually individuals with strong patent infections that show reduced allergic prevalence. Thus, further research into the immunomodulatory capacity of schistosome-egg derived factors may elucidate novel drug candidates or enhance treatment strategies to reduce allergic responses on the cellular level.

## Introduction

Over the last century and in strong contrast to third world countries, Western populations have shown a consistent rise in autoimmune disorders (e.g. Crohn's disease) and allergic conditions such as asthma [1]. Indeed, allergic asthma is the most common disease in industrialized countries, with a prevalence of up to 29% [2]. It is a potentially life-threatening illness since severe allergic responses within the respiratory airways elicit inflammation and swelling that results in varying breathing difficulties, e.g. dyspnea, tight chest, cough or bronchospasm [3]. Further clinical features of bronchial asthma include inflammatory cell recruitment and impaired lung function. Moreover, several defined immune-response characteristics can be assessed including levels of IgE specific for the allergic agent and the development of Th2 responses upon challenge with the allergen.

Epidemiological studies have clearly shown that the observed increase in allergy prevalence in Western countries is not reflected in the developing world which instigated the concept of the Hygiene Hypothesis. In essence, this hypothesis states that due to well-established sanitation and vaccination procedures, the overall reduction in common Th1-inducing (bacterial and viral) infections has resulted in a decreased ability to immunologically counterbalance Th2-driven diseases. This has created a conundrum, since a large body of epidemiological evidence and research has established that helminth diseases, which are strong Th2 inducers themselves, actually protect against developing allergic responses [4]. At first glance, the idea of protective cross-reactive mechanisms is surprising since worms drive eosinophilia and IgE production: hallmarks of pathology inducing factors in asthmatic disease. Nevertheless, after consolidating the findings of 30 independent epidemiological surveys, studying the influence of geohelminth infections on allergy prevalence, the “Parasites in Asthma Study Group” concluded that protective effects are dependent on the worm species, age, state of infection (chronic versus acute) and parasite burden [5]. Consequently, despite their Th2 inducing capacity, helminth infections are now incorporated into the “Expanded Hygiene Hypothesis”.

Interestingly, the blood fluke *Schistosoma mansoni* was one of the parasites found to have a protective effect. More than 250 million people in 74 tropical and subtropical countries are chronically infected with this trematode, which has life-stages that pass through both the skin and lung of the definite host. During the course of the disease an immune homeostasis eventually evolves that is supported by long-lasting immunomodulatory mechanisms and potentially deviates other responses. Worm development, pathology and immune responses, including the switch from Th1 to Th2 upon egg expulsion, are parallel to those seen in man and studies in mice have shown the ability of schistosomes to decrease autoimmune and allergic diseases [6]–[10]. These manipulative strategies are directed through immune cell populations such as Foxp3^+^ regulatory T cells (Treg) or B regulatory cells [11]. Treg are essential for controlling unwarranted responses to “self-antigens” [12] and during schistosomiasis this cell population increases within the CD4^+^ T cell compartment in a homeostatic fashion. Moreover, Foxp3^+^ Treg maintain granuloma development, the main cause of morbidity and develop a unique genetic signature [13], [14]. Using murine models of allergic airway inflammation (AAI), Treg in general have been shown to control overt allergic responses [15], [16] and appear to be required in mediating protection elicited via schistosome infection [17]–[19]. Here we evaluate in detail which life-cycle stage of the worm confers protection and assess the capacity of Foxp3^+^ Treg induced during infection to suppress allergic airway disease by depletion Foxp3^+^ Treg cells in the molecularly defined DEREG (Depletion in Regulatory T cell) mouse model [20].

## Methods

### Ethics statement

This animal study was conducted in accordance with an application to perform *in vivo* experiments (license number AZ. 55.2.1.54-2532-147-08) and was approved by the local government authorities Bezirksregierung Oberbayern. Animals were housed at the Institute of Medical Microbiology, Immunology and Hygiene (MIH), Technische Universität München, Germany, in accordance with the German Tierschutzgesetz (German animal protection laws) and the EU guidelines 86/809.

### Infection experiments, Treg depletion and parasitological assessment

**Figure 1:**
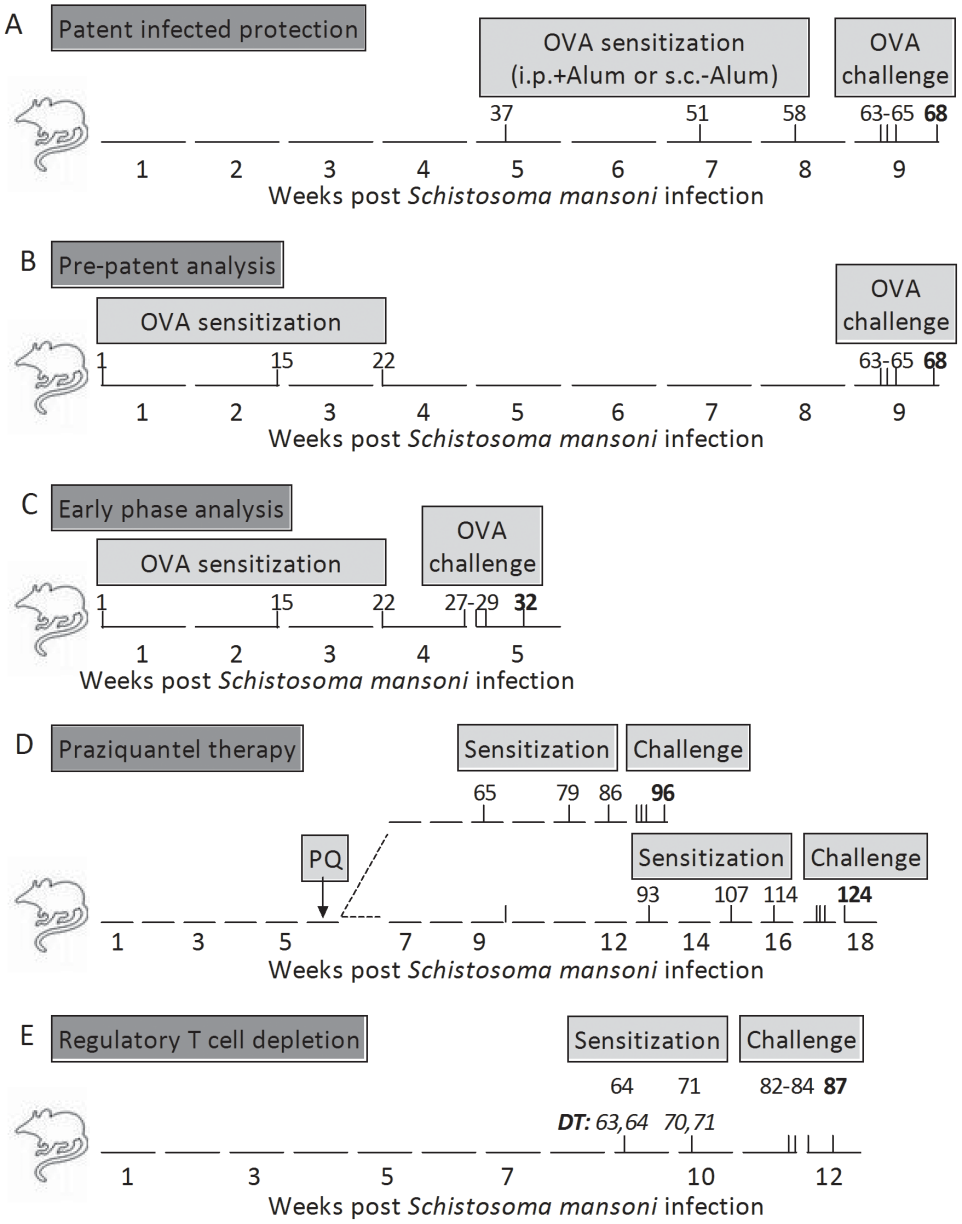
Experimental models for examining *S. mansoni*-mediated suppression of AAI. A *Patent Infected Protection (PiP)*. Groups of BALB/c female mice were infected with *S. mansoni*. Inf/OVA and OVA groups of mice were then sensitised with OVA either i.p. with Alum or s.c. without adjuvant at the indicated timepoints. Challenge occurred over three consecutive days in the 9th week of infection and analysis 5 days thereafter. B *Prepatent (PP*). Sensitizations were started in Inf/OVA and OVA groups during the first weeks of infection, before eggs are released from fecund females. Challenge and analysis were performed as in A. C *Early Phase (EP)*. AAI was performed entirely over the first 5 weeks of infection. D *Praziquantel therapy*. During the 6th week of infection, mice were orally treated with PZQ for 5 consecutive days. AAI was then begun after either 2 weeks (upper track) or 6 weeks (lower track). E *Depletion of Treg*. Groups of C57BL/6 DEREG mice were infected with *S. mansoni*. In some groups, Treg were depleted using DT injections prior to OVA sensitization at the indicated timepoints. Challenge and analysis occurred in the 12th week of infection.

Wildtype BALB/c female mice (6–8 weeks old) were purchased from Harlan (Borchen Germany). DEREG C57BL/6 mice were bred in house at the MIH. Infections with a Brazilian strain of *S. mansoni* were instigated with the injection of 90 cercariae per mouse and were performed as depicted in Figure 1A–E. Praziquantel (PZQ) was obtained from Bayer Healthcare, Leverkusen, Germany and was administered orally at a dose of 100 mg/kg body weight over 5 consecutive days during the 6^th^ week of infection (Figure 1D). *S. mansoni*-infection was confirmed through visible granuloma development in liver sections and egg burden in the liver following KOH digestion following standard techniques [21]. In the PZQ experiments, Masson's stained liver sections were also used to determine the percentage of viable eggs. In Figure 1E, gfp^+^Foxp3^+^ Treg in C57BL/6 DEREG mice were depleted by i.p. application of 1 µg diphtheria toxin (DT) purchased from Merck KGaA, Darmstadt, Germany and was dissolved in endotoxin-free PBS (PAA Laboratories GmbH, Linz, Austria) [20].

### Ovalbumin-induced allergic airway inflammation model

On the days depicted in Figure 1, intraperitoneal (i.p.) sensitizations were performed using 10 µg of grade VI ovalbumin (OVA), (Sigma, Deisenhofen, Germany) emulsified in 1.5 mg alum (aluminium hydroxide (Al[OH]_3_)) (Sigma). Subcutaneous injections of OVA (10 µg) (Figure 1A) were administered without alum at the back of the neck. Control groups were injected with PBS. Mice were challenged over three consecutive days with 10 µg of grade V OVA using the Pari-Master (PARI GmbH) aerosolic nebulizer [16]. AAI parameters were analyzed 72 hours after the last challenge. Since age has been shown to influence the intensity of developing AAI in mice [22], we limited possible age-bias by ensuring that all groups of mice were age-matched at the onset of the experiment. Moreover, they were housed for the entire experimental period under the same conditions.

### Airway Hyperresponsiveness (AHR)

Following challenge, airway responsiveness to methacholine (MCh) (Sigma) was determined in individual mice using the Flexivent system (SCIREQ, Montreal, QC, Canada). Following anaesthesia with Ketanest (Inresa Arzneimittel GmbH, Freiburg, Germany) and Rompun (Bayer Health Care, Leverkusen, Germany) mice were paralyzed with Esmeron (N.V. Organon, Oss, Netherlands). The trachea was then intubated with a 1.2 mm tracheal cannula and the lungs mechanically ventilated at a respiratory frequency of 150 breaths per min, a tidal volume of 10 ml/kg and a positive end-expiratory pressure of 3 ml H_2_O. After exposing mice to aerosolized PBS to retrieve the baseline value, bronchoconstriction was induced by increasing the concentration of MCh. Resistance was recorded over 1 minute intervals using a standardized inhalation maneuver (SnapShot-150) [23].

### Bronchoalveolar lavage and cell differentiation analysis

To obtain bronchoalveolar lavage (BAL) cells, the lungs of individual mice were rinsed with 1 ml of PBS containing proteinase inhibitor cocktail tablets (Roche Diagnostics Mannheim, Germany). The resulting fluid was weighed and centrifuged at 230 g for 5 minutes at 4°C. Cells were then re-suspended in PBS containing 2% FCS and then centrifuged (400 rpm for 5 minutes) onto glass slides using the Shandon Cytospin 3 Centrifuge (Thermo Scientific, Hamburg, Germany). After overnight drying slides were stained using the Diff-Quick staining kit (Medion Diagnostics, Langen, Germany). Cell differentiation was determined microscopically.

### Detection of OVA-specific IgE

OVA-specific IgE levels were measured in the sera of individual mice. In brief, 96-well ELISA plates (Nunc , Langenselbold, Germany) were coated overnight (4°C) with 1 µg per well of OVA grade V diluted in aqua dest. containing NaHCO_3_ and Na_2_CO_3_. After washing and blocking in 50 mM Tris solution containing 3% BSA, sera was diluted in blocking buffer (1∶200 to 1∶100,000 dilutions) and a standard of mouse α-OVA IgE antibody (Biozol, Eching, Germany) was applied in two-fold serial dilutions and incubated overnight (4°C). Subsequently, plates were washed and α-mouse IgE biotinylated detection antibody (Biozol) was applied and incubated for 2 h (RT). After further washing streptavidin-horseradish peroxidase (HRP) conjugate (R&D Systems GmbH, Wiesbaden, Germany) was added and plates were incubated for 30 min (RT) in the dark. After a final washing step, TMB substrate (BD, Heidelberg, Germany) was applied and reactions were stopped with 2MH_2_SO_4_. Finally, ODs were determined at 450 nm using the Sunrise ELISA microplate reader (Tecan, Crailsheim, Germany). The concentration of the samples was then calculated according to the standard curve.

### Lung pathology and immunohistochemistry

Paraffin embedded sections (3 µm) from the left lungs of individual mice were stained with PAS (Periodic acid-Schiff). Lung tissue inflammation was microscopically determined by the degree of cell infiltration around the basal membrane of bronchi or vessels, which was graded on a scale from 1 to 4. In brief, a value of 1 was assigned for occasional cuffing with inflammatory cells, a value of 2 was assigned for a thin layer (two to three cells thick) of inflammatory cells, a value of 3 was assigned when bronchi or vessels were surrounded by a thick layer of four to five inflammatory cells and a value of 4 was assigned when bronchi or vessels were surrounded by a layer of more than five inflammatory cells. Per lung section, a mean inflammation score was determined. Immunohistochemistry of CD3^+^Foxp3^+^ Treg was performed as previously described [14]. In brief, lung sections were deparaffinized and heated in sodium citrate buffer (pH6.0) for 2 min at high pressure. After washing and blocking, slides were incubated with a goat polyclonal antibody (Ab) against the C terminus of the Foxp3 protein (ab2481, dilution 1∶50; Abcam Limited, Cambridge, U.K.). Sections were then exposed to a biotin-conjugated rabbit anti-goat and the EnVision peroxidase kit (Dako-Cytomation). After a further incubation with the second Ab against CD3 (clone UCHT1, 1∶25; DakoCytomation) cells were visualized using the alkaline phosphatase–anti-alkaline phosphatase method [24]. In a blind fashion, Foxp3^+^ T cells were quantified in individual lung sections within a 1 mm^2^ area using a Zeiss microscope (Axio Lab.A1, Oberkochen, Germany) at 20× magnification.

### Flow cytometry staining of Treg

Intracellular staining of Foxp3^+^CD4^+^ T cell populations was performed on erythrocyte-depleted mediastinal lymphnode cells (medLN). Prior to staining, Fc receptors were blocked using anti-CD16/CD32 (eBiosciences, Frankfurt, Germany). Thereafter, cells were stained with APC-conjugated anti-CD4 mAb (eBiosciences). Intracellular Foxp3 levels were detected using the PE-conjugated anti-Foxp3 mAb staining kit according to the manufacture's instruction (eBiosciences). Fluorescence was analyzed using a flow cytometer and software from BDbiosciences (Heidelberg, Germany). The presence or absence of egfp^+^ cells in DEREG mice was also detected by flow cytometry in peripheral blood samples from individual mice [20].

### *In vitro* re-stimulation

Erythrocyte-depleted cell suspensions (2×10^5^) from individual lung lymph node (LLN) or individual spleens were re-stimulated *in vitro* for 72 hours with 10 µg/ml OVA (Grade VI) or with 20 µg/ml soluble egg antigen (SEA) prepared from schistosomal eggs as previously described [13]. Cytokine content in the culture supernatant was then determined using ELISA in accordance with the manufacturer's instructions (eBiosciences).

### Statistical analysis

Statistical differences were analyzed using GraphPad Prism 5 software (San Diego, CA, USA). Parametrically distributed data were analyzed using unpaired t-tests or one-way ANOVA.

## Results

### Patent infections of *S. mansoni* reduce allergic airway inflammation

**Figure 2:**
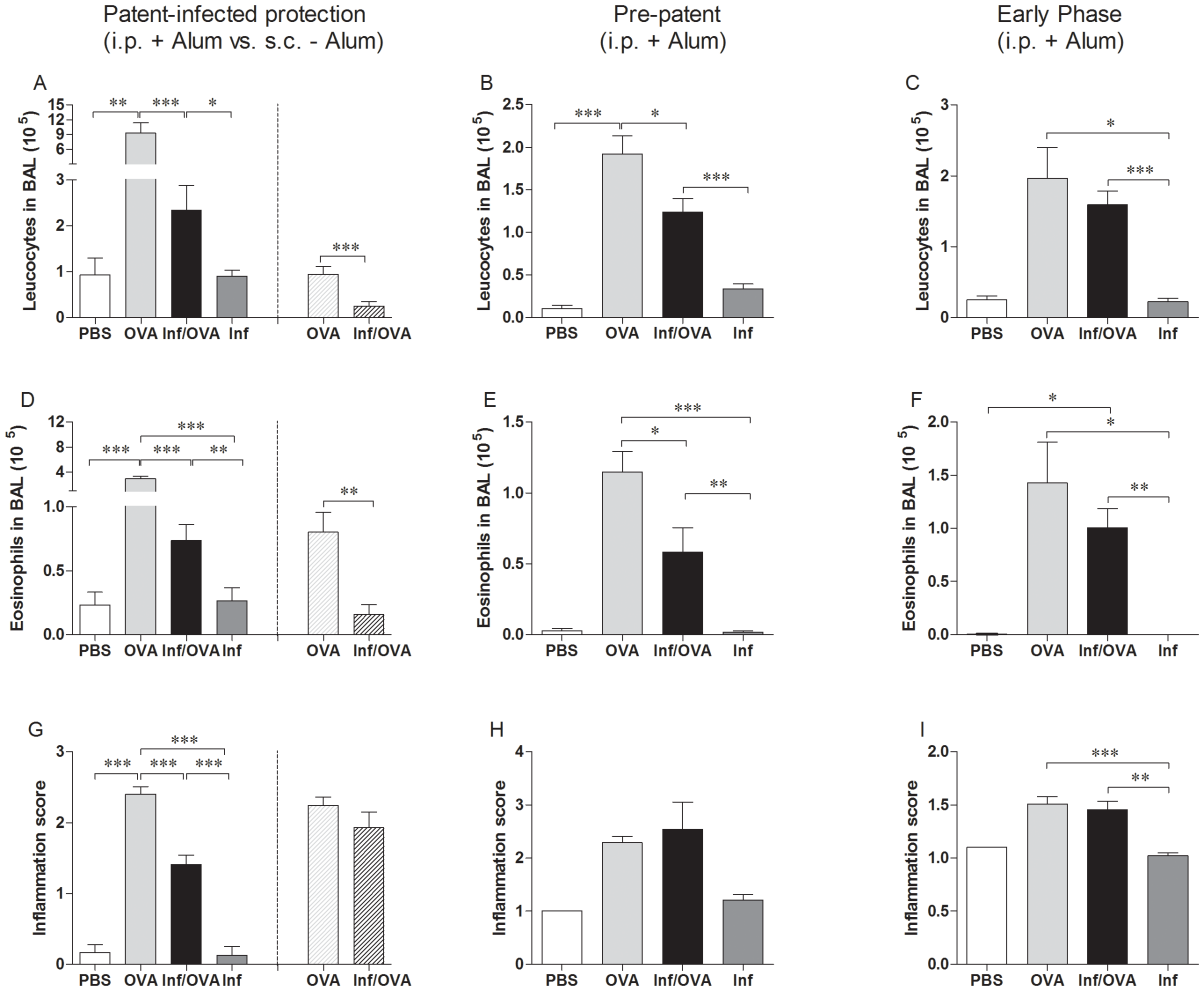
Suppression of AAI in *S. mansoni*-infected mice requires patency. Groups of BALB/c female mice were infected with *S. mansoni*. Left PiP experiments: After the 5th week of infection, OVA sensitizations were administered either i.p. with alum or s.c. in the absence of adjuvant (Figure 1A). Middle PP experiments: sensitizations were given during the first weeks of infection (before patency) but challenge was performed at the same time-point of infection as in PiP investigations (Figure 1B). Right EP studies: both sensitizations and challenge were performed before the onset of patency (Figure 1C). Upon AAI analysis, individual mice were assessed for leucocyte numbers (A–C) and eosinophil levels (D–F). Lung sections were also scored for inflammation (G–I). In i.p.+Alum experiments displayed in A, D and G, bars show mean + SD of individual mice from 5 independent infection/allergy experiments (PBS, n = 21; OVA, n = 34; Inf/OVA, n = 29 and Inf., n = 28). In s.c.-Alum experiments OVA, n = 8 and Inf/OVA, n = 7. In PP experiments B, E and H, bars show mean + SD of individual mice from 2 independent infection/allergy experiments (PBS, n = 2; OVA, n = 14; Inf/OVA, n = 13 and Inf., n = 10). In EP experiments C, F and I, bars show mean + SD of individual mice from 1 or 3 similar independent infection/allergy experiments (PBS, n = 2; OVA, n = 10; Inf/OVA, n = 10 and Inf., n = 4). Asterisks show statistical differences (ANOVA or Student's t test) between the groups indicated by the brackets (*p<0.05, **p<0.01, ***p<0.001).

Although humans are the main definite host of *S. mansoni* in many regions, this helminth also infects rodents. Moreover, immunological and pathological responses observed in humans are mirrored in mice. Worms require 5–6 weeks to mature and enter patency which is classified as the production of eggs by fecund females [25]. Previous studies have shown that an ongoing infection dampens AAI in C57BL/6 mice [7]. Using the Th2 biased BALB/c mouse strain [26], [27] we aimed to differentiate which stage of infection provided a protective environment and performed three different infection/allergy scenarios which are depicted in Figures 1A–C. To confirm the onset of asthma numerous parameters were assessed including cell infiltration levels in the BAL (Fig. 2A–F), lung inflammation (Figures 2G–I and S1A–D) and AHR (Figure S1E). First, to observe whether patently-infected BALB/c mice were protected against the development of AAI, mice were sensitized on days 37, 51 and 58 post-infection (Figure 1A - PiP: patent-infected-protection). Sensitizations were administered either i.p. in the presence of alum or s.c. in the absence of adjuvant. Mice sensitized and challenged during patent infection (Inf/OVA) had significantly decreased cell infiltration (Figure 2A) and eosinophil numbers (Figure 2D) when compared to non-infected OVA groups (OVA), indicating that *S. mansoni* infections in BALB/c mice protect against allergy development. These findings were also observed using the adjuvant-free model (Figure 2A right and D right). Within the lung, decreased inflammation was only observed in Inf/OVA groups that had been sensitized with alum (Figure 2G). Figures S1A–D show representative histological sections from the different groups of mice in these PiP experiments. As shown, when compared to OVA groups (Figure S1A), inflammation was less severe in Inf/OVA groups (Figure S1B). Control groups that were (Inf.) or were not infected (PBS) and only exposed to aerosolic OVA upon challenge did not present any signs of inflammation or cell infiltration (Figures 2A–I and S1C and D). Airway resistance was also measured in the PiP investigation studies and as with the other measured parameters, Inf/OVA mice had resistance levels that were comparable to the control groups (Figure S1E). With regards to immunological responses, OVA-specific Th2 (Figures S2A and B) and regulatory (Figure S2C) recall responses of draining lymph node cells were significantly dampened in Inf/OVA groups and this was regardless to the sensitisation route. Interestingly, OVA-specific IgE levels in the sera were elevated in Inf/OVA mice that were sensitised i.p. but not s.c. (Figure S2D).

Next, we assessed whether protection occurred when infected mice received sensitization rounds within the first 21 days of infection (prepatency) but were challenged during patency (Figure 2B - PP: prepatent). Interestingly, although leucocyte and eosinophil infiltration was still significantly dampened (Figures 2B and E respectively), within this experimental scenario, no protective influence on lung inflammation could be observed in the Inf/OVA group (Figure 2H). Finally, experiments were performed in which AAI was induced and assessed during prepatency i.e. the first five weeks of infection (Figure 2C - EP: early phase). Here, *S. mansoni* infection elicited no protection since cellular infiltration and lung inflammation scores were equal to OVA groups (Figures 2C, F and I). Parasitological assessment of the *S. mansoni* groups, in all infection scenarios, showed no differences between granuloma size or egg burden in the liver (data not shown). Eggs were not detected in the EP experiments since at week 4–5, adult female worms are not yet fecund.

In another set of experiments we investigated the development of AAI upon curing a patent *S. mansoni* infection (Figure 1D). In brief, groups of infected mice were treated with praziquantel (PZQ) during the 6^th^ week of infection. After a further 2 or 6 weeks, OVA sensitization commenced, thus, analysis of AAI was performed after either 12 or 18 weeks post infection (or 6 and 10 weeks post PZQ-therapy). Interestingly, infected PZQ-treated groups of mice (Inf/OVA/PQ) analysed after 12 weeks but not 18 weeks post-infection were still significantly protected from cellular infiltration (Figures S3A–D) but not inflammation (Figures S3E and F). As shown in Figure S3G, egg counts in the liver were reduced after 10 weeks of PZQ therapy. This was also confirmed in the amount of released eggs in the stool since none were detected on the days of analysis (data not shown). Moreover, upon assessment of individual liver sections, there was a significant reduction in viable eggs in the liver after 10 weeks of PZQ treatment (Figure S3H).

### Absence of Foxp3^+^ Treg in lung sections of Inf/OVA mice

**Figure 3:**
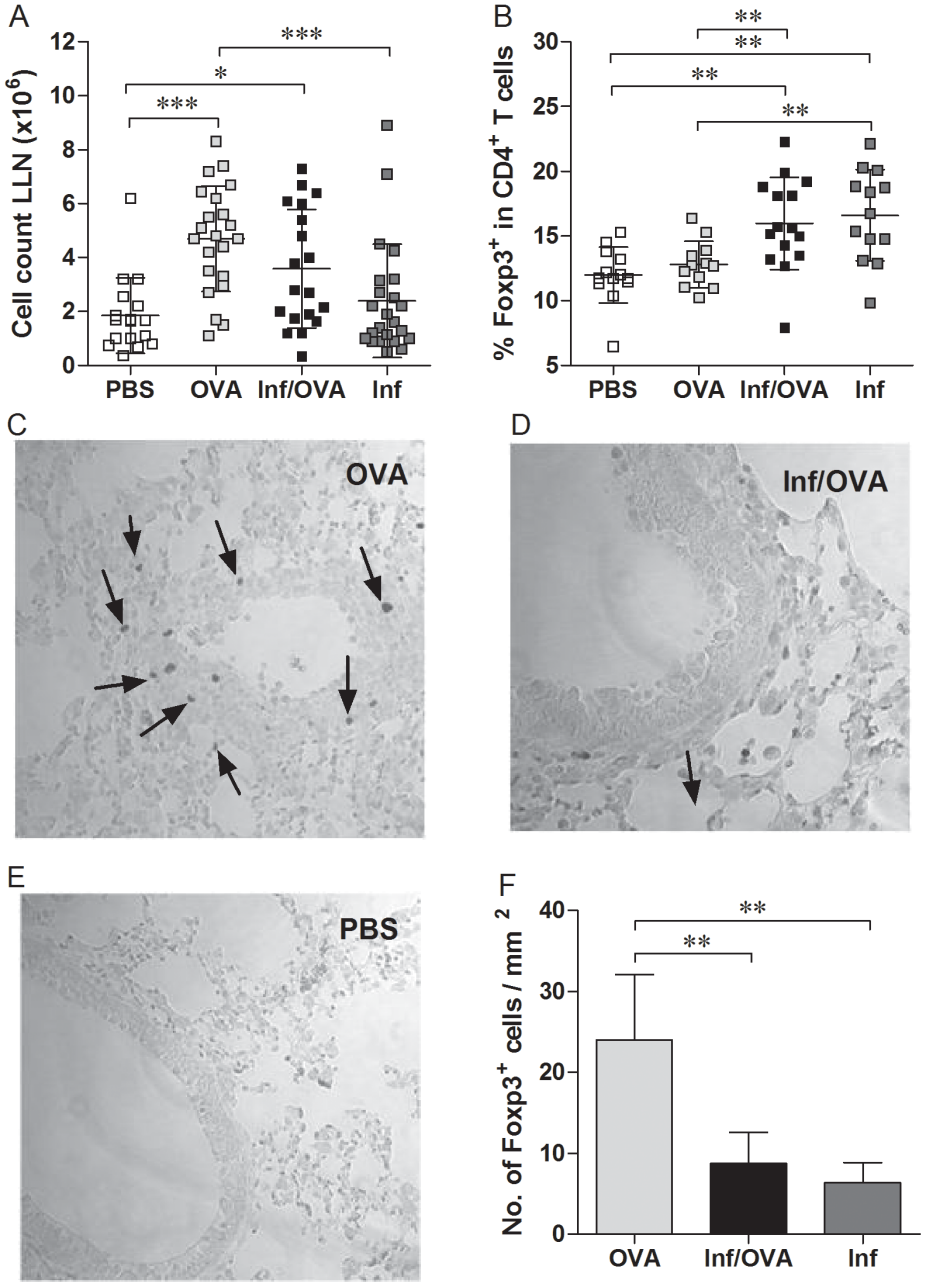
Increased Foxp3^+^ Treg frequency in the draining lymph nodes of Inf/OVA mice is not reflected in lung sections. A) Total cell counts and B) the number of CD4^+^Foxp3^+^ T cells in the draining lymph nodes of individual mice on the day of analysis in PiP studies. Symbols depict the levels in each mouse and data shows values from 5 independent infection studies (PBS, n = 21; OVA, n = 34; Inf/OVA, n = 29 and Inf., n = 28). CD3^+^Foxp3^+^ T cells in the lung sections of C) OVA mice, D) Inf/OVA mice and E) control groups (PBS) were determined by immunohistochemical staining. Arrows depict positive cells. F) Quantification of CD3^+^Foxp3^+^ T cells in individual lung sections was determined within a 1 mm^2^ area. Asterisks show statistical differences (ANOVA) between the groups indicated by the brackets (*p<0.05, **p<0.01, ***p<0.001).

As mentioned above, previous studies have shown that Treg play a role in helminth-mediated immunomodulation during AAI. However, questions pertaining to their redistribution into the lymphatics or relevant organ tissues remain unanswered. Thus, we analysed the levels and distribution of Foxp3^+^ T cells in the draining lymph nodes (LLN) of mice from the PiP experiments (Figure 2A). Figure 3A shows that the absolute cell counts in OVA treated mice, regardless of infection, is higher than in PBS groups. No significant differences could be observed between OVA and Inf/OVA groups. With regards to the percentage of Foxp3^+^ T cells in the CD4^+^ T cell compartment, these regulatory cells were elevated in Inf/OVA mice but not in OVA groups alone (*c.f* OVA and Inf/OVA in Figure 3B). Interestingly, Treg numbers were high in the Inf. group as well. Within the lung however, numerous CD3^+^Foxp3^+^ T cells could be identified in sections from OVA mice (Figure 3C) but not Inf/OVA groups (Figure 3D). These sections were comparable to those sampled from the PBS groups (Figure 3E). These impressions were verified through further quantification of the Treg numbers in the lung sections of individual mice. As shown in Figure 3F, CD3^+^Foxp3^+^ Treg were significantly higher in OVA but not Inf/OVA group.

### Lack of Treg during sensitization impedes *S. mansoni*-mediated suppression of AAI

**Figure 4:**
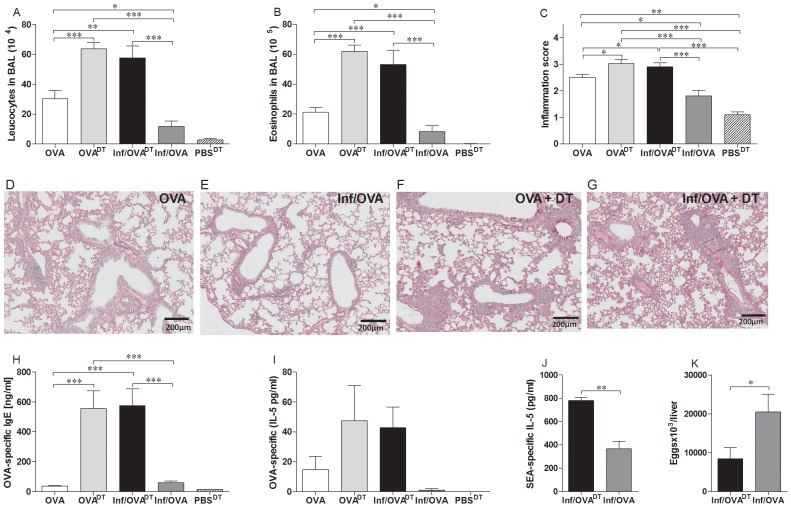
Depletion of Treg during sensitization reverts schistosome-mediated dampening of AAI. As depicted in Figure 1F, groups of C57BL/6 DEREG mice were infected with *S. mansoni*. On the 9^th^ and 10^th^ week of infection mice were sensitized i.p. with OVA in the presence of alum. Prior to sensitization, Treg were depleted by the administration of DT. During the 12th week of infection, mice were challenged and assessed for the number of leucocytes (A) and eosinophils (B) in the BAL. Levels of inflammation were also scored using lung sections stained with PAS (C–G). OVA-specific IgE levels were measured in the sera of individual mice via ELISA (H). OVA-specific responses were measured following stimulation of spleen cells (2×10^5^) with 10 µg/ml OVA (I). In addition, schistosome-specific responses were evaluated upon re-stimulation of spleen cells with SEA (25 µg/ml) (J). After 72 hours, IL-5 levels were determined in the culture supernatant by ELISA (I and J). Egg burden was measured in livers of individually infected mice (K). Bars show data from one of two similar experiments (OVA, n = 7; OVA^DT^, n = 7; Inf/OVA^DT^, n = 5; Inf/OVA n  = 8; PBS^DT^ n = 3). Asterisks show statistical differences (ANOVA or Student's t test) between the groups indicated by the brackets (*p<0.05, p<0.01, ***p<0.001).

To address whether *S. mansoni*-mediated protection of AAI was mediated by Treg we infected DEREG mice, which allow the specific depletion of egfp^+^Foxp3^+^ cells at specified time-points through the administration of DT [20]. Initially we performed such depletion experiments in BALB/c DEREG mice but due to their higher sensitivity to DT application and schistosome infection, Treg depletion during AAI development resulted in high mortality rates (over 90%, data not shown). Therefore, we continued the experiments with DEREG mice on a C57BL/6 background. As depicted in Figure 1E, Treg were depleted in groups of infected and non-infected mice during sensitization. As observed before within the PiP studies described above, Inf/OVA groups had significantly lower levels of leucocytes and eosinophils when compared to OVA groups (*c.f.* bars 1 and 4 in Figures 4A and B). However, depletion of Treg in the infected group (Inf/OVA^DT^) prevented the previously observed suppressive effects (*c.f.* bar 3 with bars 1 and 4 in Figures 4A and B) and levels of cellular infiltration even equalled those of the OVA^DT^ groups. In association with the current literature [16], non-infected OVA groups, depleted of Treg, also showed increased leucocyte and eosinophil infiltration(*c.f.* bars 1 and 2 in Figures 4A and B). With regards to inflammation score depletion of Treg abrogated the suppressive effect of schistosome infection (Figure 4C *c.f*. bars 3 and 4 and Figures 4E and G). Moreover, Treg depletion led to enhanced lung inflammation when compared with the OVA group (Figures 4C *c.f* bar 1 with 2 and 3 and Figures 4D,4F and 4G). Immunologically, both Treg depleted groups showed significantly higher levels of OVA-specific IgE in the sera (Figure 4H) and OVA-specific IL-5 upon recall of splenocytes cells (Figure 4I). Interestingly, schistosome-specific responses in the infected groups were also significantly increased in the Inf/OVA^DT^ groups (Figure 4J). Moreover, these mice showed lower egg burden in the liver (Figure 4K). These data correlate to our earlier studies in which we depleted Tregs in *S. mansoni* infected mice by administering an anti-CD25 antibody. There, we also observed elevated schistosome-specific Th2 responses and strongly decreased egg numbers in the livers of Treg-depleted *S. mansoni* infected animals [13]. During these experiments, we controlled for the depletion of Treg by observing levels of CD4^+^egfp^+^ T cells in peripheral blood. Figure S4A shows a representative comparison of CD4^+^egfp^+^ T cells in infected (left) and non-infected (right) mice on the day before depletion and 3 days thereafter. As shown in the bottom panel of flow cytometry images, no egfp^+^ cells are visible after depletion. Levels were measured again following asthma induction (Figure S4B). Here, the percentage of Foxp3^+^ Treg in infected-depleted mice was approximately 50% lower than in non-infected depleted mice (4.7% vs 8.24%). This was observed in all the experimental mice (Figure S4C) and indicates that the re-establishment of Treg in infected mice is slower than in non-infected controls and provides a possible explanation as to why infected mice are no longer suppressed from AAI development.

## Discussion

Schistosome infections result in immune responses that are tightly controlled by various host mechanisms including cell types such as Treg and Breg [13], [28] or elicited through the release of immunosuppressive schistosomal antigens [29]. The latter is thought to generate bystander immune responses against unrelated antigens or allergens. Several studies have addressed the role of helminth infections using the classical ovalbumin model of AAI. With regards to schistosome-allergy studies both protective and aggravated responses have been noted [7], [19], [30]. For example, Smits *et al*. showed that only chronically infected C57BL/6 mice (12–16 weeks) were protected from AAI, whereas in our study protection in BALB/c mice was already observed at 9 weeks of infection. Our studies required the induction of AAI at this time-point since in contrast to C57BL/6 mice, BALB/c mice have an accelerated disease progression and higher mortality rate. Our findings are further supported by the studies from Pacifico *et al.* who reported a protective effect in BALB/c mice, also at 9 weeks of infection, using a dose of OVA and alum that was equivalent to that employed in our study [19]. This is in contrast to Mangan *et al*., who used double the amount of OVA and alum and interestingly, they reported aggravated airway hyper-responsiveness and enhanced OVA-specific Th2 responses in acute and chronically schistosome-infected BALB/c mice [30]. Perhaps such strong immunizations can no longer be counterbalanced by the schistosome infection. These opposing studies clearly highlight that the different protocols used in the field trigger different immune responses and have to be considered when interpreting and comparing the results. Interestingly, in the Mangan study infections with male schistosome worms resulted in reduced airway responses which was mediated through B cells and IL-10 but not Treg. Hypothetically therefore, worm-induced B cells and egg induced Treg could both contribute to suppression of AAI. Another factor which has to be stringently addressed is age, since it is known that age effects the development of AAI in mice [22]. To ensure that our findings were not due to an age-bias, all mice were age–matched at the onset of experiments and housed under the same conditions.

Evidently, *S. mansoni*-infected mice had reduced AAI when sensitized and challenged during patent infection and moreover, Inf/OVA mice still had dampened responses when challenge but not sensitization occurred in the patent phase. However, no protection was observed in Inf/OVA mice when both sensitization and challenge were performed in the prepatent phase (EP studies) or six weeks after praziquantel treatment. Taken together these results clearly highlight the requirement of patency for infection-mediated AAI suppression. Interestingly, our anti-helminthic treatment experiments revealed that some level of protection against AAI remained when sensitization commenced two weeks after treatment. At this time-point although many tissue-residing eggs are still viable, most of the worms have died and only stunted, non-egg producing worms are left over [31], [32]. Complete abrogation of the protective effects was only observed in those experiments analyzed on the 18^th^ week of infection, when sensitization began 6 weeks after PZQ treatment. Here it is considered that the proportion of viable eggs has strongly decreased and our findings here coincide with these data [13]. In association, a human study demonstrated that schistosome-infected individuals displayed increased levels of HDM-specific IgE and low responses to skin prick tests [33]. However, following anti-helminthic treatment these patients presented increased HDM skin reactivity [34]–[36] indicating that in order to maintain a suppressive milieu an ongoing patent infection is required. The patently-induced protection was also independent of the OVA-application route and the presence of alum. OVA-specific IgE levels in infected mice were increased when OVA was injected i.p. with alum, which has been reported to drive the induction of the disease independently from mast-cell mediated early phase reactions [37], [38]. OVA-specific IgE levels remained unaltered when alum was applied subcutaneously which indicates that the suppression of AAI occurs independently of IgE levels. Thus, we conclude that the suppressive effects mediated by schistosome infection are not due to defective OVA priming which supports earlier findings [30].

Concerning the possible underlying mechanisms that lead to dampened allergic responses in schistosome infected animals, we and others have shown previously that Foxp3^+^ Treg expand homeostatically during the course of infection and this phenomenon already starts when the first SEA-specific Th1 responses can be detected around the 5^th^ week of infection [13]. The first hint that Treg might be involved in schistosome-mediated protection of AAI came from the finding that when compared to OVA groups, Foxp3^+^ Treg were significantly elevated in the LLN of Inf/OVA and Inf alone groups of mice. However, whereas numerous CD3^+^Foxp3^+^ Treg were present in the lung tissues of OVA groups very few were detectable in Inf/OVA mice. Therefore, we consider that during schistosome infection, regulation of lung infiltrate upon challenge occurs at the draining LLN and not within the lung tissue itself. It will be interesting to decipher how Treg in Inf/OVA mice are retained in the LLN and whether these are indeed infection-induced Foxp3^+^ T cells. In our previous studies we showed that infection-induced Treg but not Treg from naive mice suppress SEA-specific CD4^+^ T cell effector responses and develop a unique gene expression profile that includes upregulation of granzyme B and anti-inflammatory molecules such as SLPI (secretory leucocyte peptidase inhibitor) [14]. This change of phenotype could account for their selective suppressive activity since it was shown, for example, that activated Treg cells can actually kill B cells in a granzyme-B-dependent manner [39]. It is now discussed that Treg adapt their mode of immune suppression according to the altered requirements found under inflammatory conditions in comparison to those in the steady-state [12]. The role of these molecules in Treg-mediated suppression of allergies *in vivo* will be an interesting field of future research.

To assess whether Treg played a role in the development of AAI during infection we employed the DEREG mouse model which allows depletion of Treg at the experimenter's desired time-points [20], [40]. This model ensures that all Foxp3^+^ T cells are depleted regardless of their CD25-coexpression and also circumvents the criticized application of anti-CD25 antibodies in which recently activated effector T cells are targeted as well [20], [41], [42]. BALB/c mice have a higher morbidity due to schistosome infection and additional DT application further increased mortality rates. Thus, we changed to the DEREG mice on a C57BL/6 background, which tolerated our experimental procedure [20]. Interestingly, Treg depletion during OVA-sensitization in C57BL/6 DEREG mice resulted in a loss of protection in *S. mansoni* infected mice. Furthermore, cellular infiltration levels and pathology scores were higher in Inf/OVA^DT^ groups when compared to control OVA groups. These findings confirm other studies that have shown the requirement for Treg in AAI [16] and that Foxp3^+^ T cells down-modulate airway eosinophilia but not AHR and IgE levels in a model of SIT-induced tolerance [43]. Indeed, OVA-specific IgE levels were enhanced in both depleted groups indicating a role of Treg in initiating Th2 responses. In addition, schistosome-specific immune responses were elevated in Inf/OVA^DT^ mice and alongside the strongly reduced egg burden this confirms our previous findings in schistosome-infected mice treated with anti-CD25 antibody [13]. In addition, we noted a delayed reconstitution of expanding Treg in Inf/OVA^DT^ mice compared to OVA^DT^ mice after Treg depletion. Thus, the removal of Treg during a chronic infection, in contrast to the steady-state or immunization situation, probably gives rise to more pronounced and quicker Th responses against schistosome antigens. This might favor immune reactions against OVA antigens and eventually results in decelerated or unbalanced Treg reconstitution.

Collectively, by comparative experiments using different protocols for AAI our data support recent findings that schistosome infection can indeed suppress allergic airway responses and that this suppression requires patency and the continuous release of eggs. Furthermore, this suppression is partly mediated by expanding Foxp3^+^ Treg cells within the draining lymph nodes of the lung. We therefore propose the concept that patent infection inhibits the effector phase of AAI through schistosome egg-derived factors and not worm or schistosomula antigens. An in-depth analysis of egg components and their potential to drive Treg expansion or induction could therefore have potential therapeutic value.

## Supporting Information

Figure S1 Lung pathology and airway resistance in PiP investigations. Groups of BALB/c mice were infected with *S. mansoni*. During the 6th to 8th week of infection, mice were thrice sensitised i.p. with OVA and Alum. Following aerosolic OVA challenge lung sections from individual mice were assessed for their level of inflammation. Representative PAS stained lung sections from OVA, Inf/OVA, PBS control and infected alone groups of mice are depicted in A–D respectively and in E airway resistance from groups of mice in PiP investigations. Symbols show mean + SD of each group of mice (n = 12/group). Asterisks show significant differences between the groups indicated by the brackets (**p<0.01).
(TIF)

Figure S2 *S. mansoni* infected mice present suppressed OVA-specific Th responses and IgE levels upon the development of AAI. Groups of BALB/c mice were infected with *S. mansoni* according to the PiP investigation protocol. During the 6th to 8th week of infection, mice were thrice sensitised either i.p. with OVA and Alum or s.c. with OVA alone. Following challenge, erythrocyte-depleted LLN cells (2×10^5^ per well) were re-stimulated *in vitro* with OVA (10 µg/ml) for 72 hours. Culture supernatants were then screened for their content of A) IL-5, B) IL-13 or C) IL-10 by ELISA. D) OVA-specific IgE levels were measured in the sera of individual mice. Bars depict mean + SD. Asterisks show statistical differences (Student's t test) between the groups indicated by the brackets (*p<0.05, **p<0.01).
(TIF)

Figure S3 Worm elimination through praziquantel therapy reverts protection against AAI. Groups of BALB/c mice were infected with *S. mansoni*. During the 6^th^ week of infection, schistosomes were killed by the oral administration of praziquantel (100 mg/kg body weight) over 5 consecutive days. i.p. OVA sensitizations commenced after either 2 or 6 weeks post PZQ treatment (Figure 1D). Groups of non-infected OVA mice were maintained under the same conditions throughout the experiment. A–F shows the changes in AAI parameters in mice analyzed after 6 (A, C and E) or 10 (B, D and F) post PZQ therapy. Graphs show leucocyte infiltration (A and B), eosinophil number (C and D) and inflammation score (E and F) in lungs of individual mice. G shows egg counts in the liver following digestion with KOH. H depicts number of viable eggs in individual livers following assessment with Masson's stained liver sections. Bars show mean ± SD from one of two experiments containing 6–8 mice per group. Asterisks show statistical differences (ANOVA) between the groups indicated by the brackets (*p<0.05, **p<0.01).
(TIF)

Figure S4 Effective Treg depletion but reduced recovery of Foxp3^+^ T cells in *S. mansoni* infected DEREG mice upon asthma induction. In A) the efficacy of Treg depletion was controlled by analyzing the percentage of cells in peripheral blood by flow cytometry (see Figure 1E). In brief, prior to depletion (d50, upper panel) and 3 days after DT injections (d53, lower panel) the percentage of CD4^+^egfp^+^ T cells was observed in *S. mansoni* infected (left) and naive DEREG mice (right). B) Upon asthma induction, the percentage of Treg was observed again in peripheral blood (d74). Representative dot plot on the left depicts the levels of CD4^+^egfp^+^ T cells in a Inf/OVA^DT^ mouse and the right image those observed in a OVA^DT^ mouse. C) Bars represent the mean + SEM of CD4^+^egfp^+^ T cells on d74 recovered from 4–5 mice per group. Percentages were calculated by flow cytometry. Asterisks show statistical differences (Student's t test) between the groups indicated by the brackets (**p<0.01).
(TIF)
