# Supplementary figures and images for: Schistosoma mansoni-Mediated Suppression of Allergic Airway Inflammation Requires Patency and Foxp3+ Treg Cells
Source: PLoS Negl Trop Dis. 2013 Aug 15;7(8):e2379. doi: 10.1371/journal.pntd.0002379 (PMC3744427; doi:10.1371/journal.pntd.0002379)

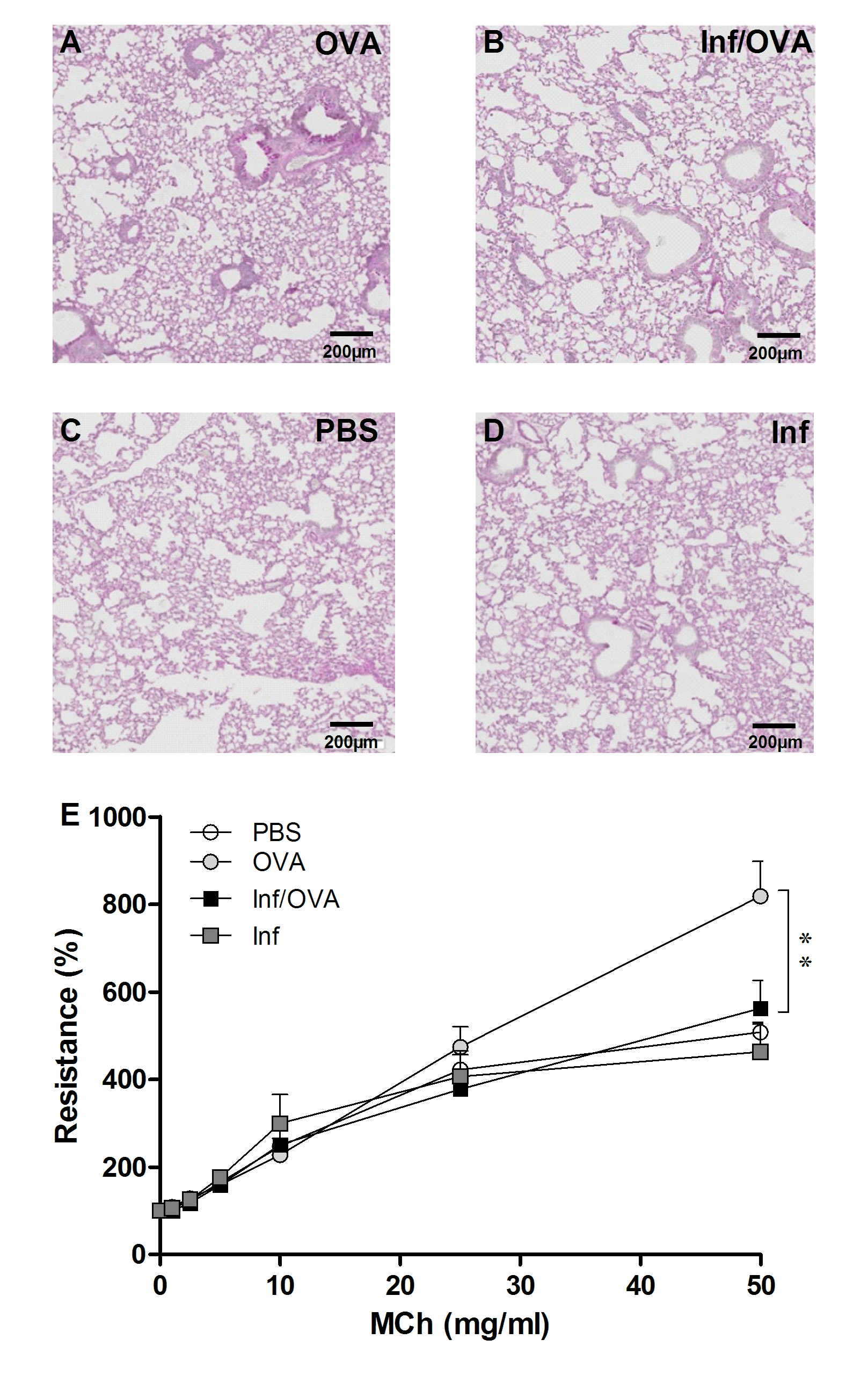

Supplement: Figure S1 — Lung pathology and airway resistance in PiP investigations. Groups of BALB/c mice were infected with S. mansoni. During the 6th to 8th week of infection, mice were thrice sensitised i.p. with OVA and Alum. Following aerosolic OVA challenge lung sections from individual mice were assessed for their level of inflammation. Representative PAS stained lung sections from OVA, Inf/OVA, PBS control and infected alone groups of mice are depicted in A–D respectively and in E airway resistance from groups of mice in PiP investigations. Symbols show mean + SD of each group of mice (n = 12/group). Asterisks show significant differences between the groups indicated by the brackets (**p<0.01). (TIF) [file pntd.0002379.s001.tif]

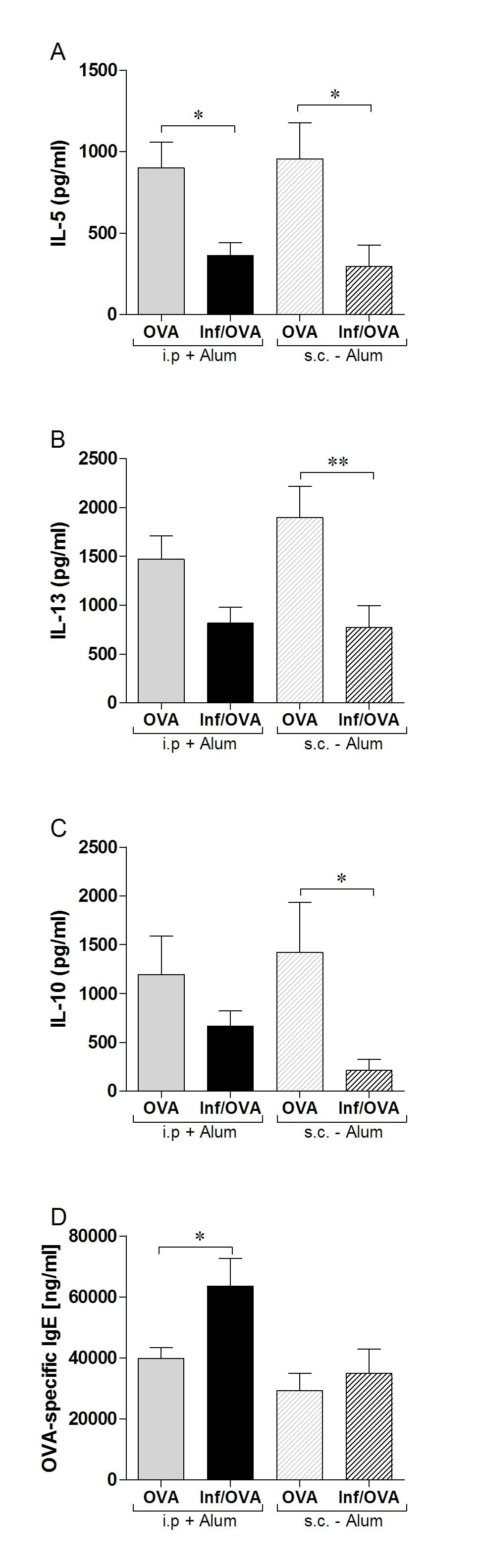

Supplement: Figure S2 — S. mansoni infected mice present suppressed OVA-specific Th responses and IgE levels upon the development of AAI. Groups of BALB/c mice were infected with S. mansoni according to the PiP investigation protocol. During the 6th to 8th week of infection, mice were thrice sensitised either i.p. with OVA and Alum or s.c. with OVA alone. Following challenge, erythrocyte-depleted LLN cells (2×10^5^ per well) were re-stimulated in vitro with OVA (10 µg/ml) for 72 hours. Culture supernatants were then screened for their content of A) IL-5, B) IL-13 or C) IL-10 by ELISA. D) OVA-specific IgE levels were measured in the sera of individual mice. Bars depict mean + SD. Asterisks show statistical differences (Student's t test) between the groups indicated by the brackets (*p<0.05, **p<0.01). (TIF) [file pntd.0002379.s002.tif]

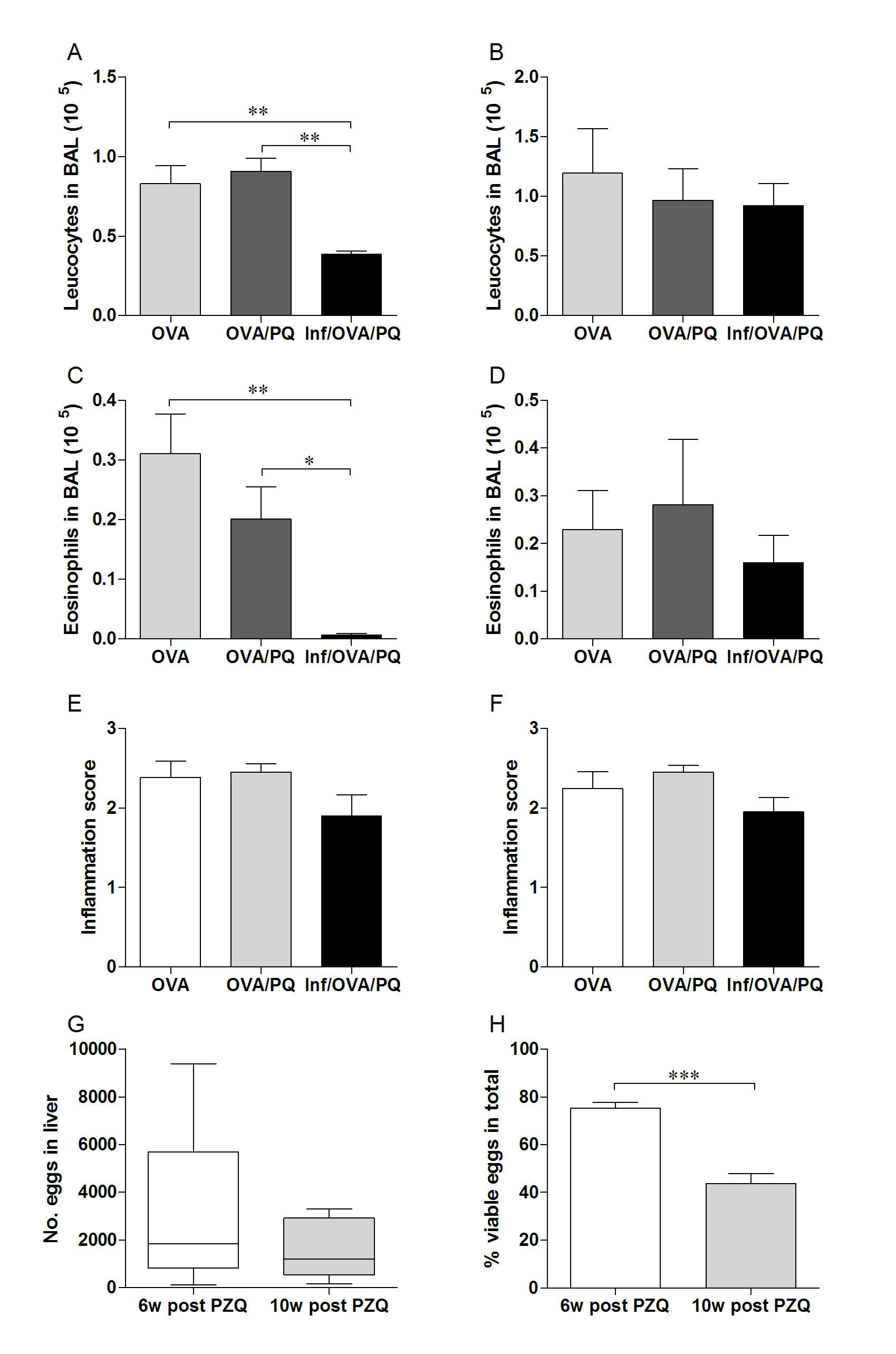

Supplement: Figure S3 — Worm elimination through praziquantel therapy reverts protection against AAI. Groups of BALB/c mice were infected with S. mansoni. During the 6th week of infection, schistosomes were killed by the oral administration of praziquantel (100 mg/kg body weight) over 5 consecutive days. i.p. OVA sensitizations commenced after either 2 or 6 weeks post PZQ treatment (Figure 1D). Groups of non-infected OVA mice were maintained under the same conditions throughout the experiment. A–F shows the changes in AAI parameters in mice analyzed after 6 (A, C and E) or 10 (B, D and F) post PZQ therapy. Graphs show leucocyte infiltration (A and B), eosinophil number (C and D) and inflammation score (E and F) in lungs of individual mice. G shows egg counts in the liver following digestion with KOH. H depicts number of viable eggs in individual livers following assessment with Masson's stained liver sections. Bars show mean ± SD from one of two experiments containing 6–8 mice per group. Asterisks show statistical differences (ANOVA) between the groups indicated by the brackets (*p<0.05, **p<0.01). (TIF) [file pntd.0002379.s003.tif]

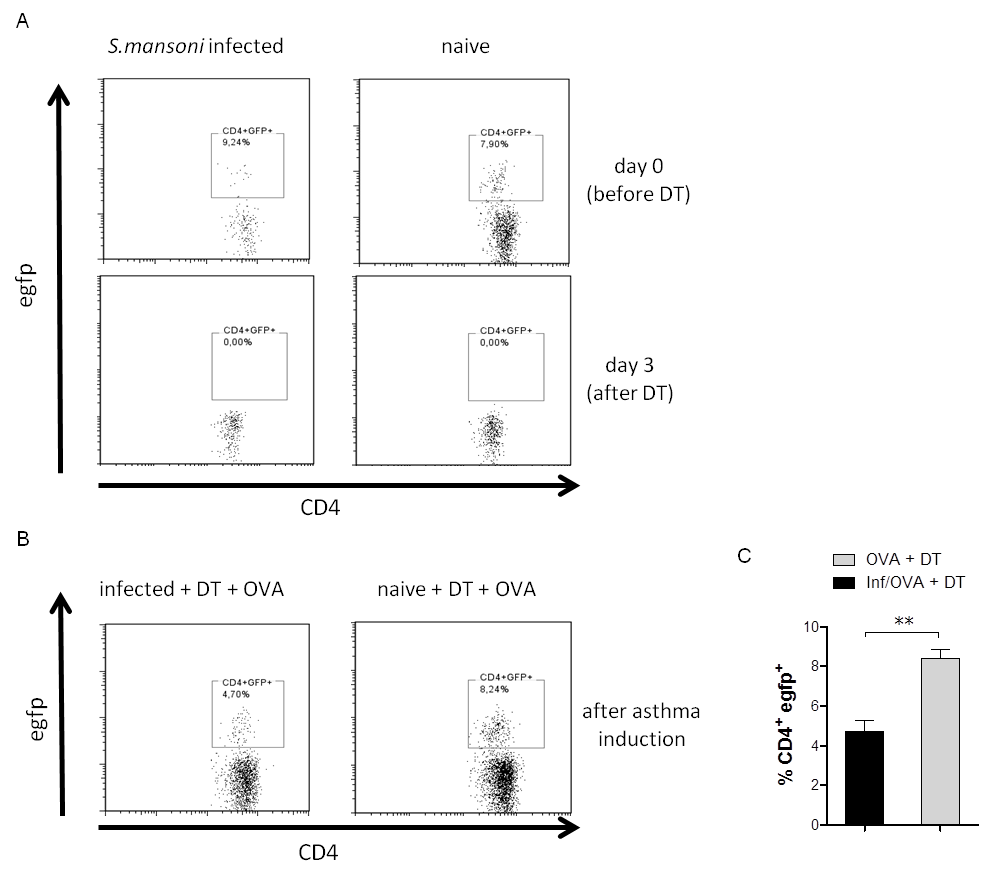

Supplement: Figure S4 — Effective Treg depletion but reduced recovery of Foxp3+ T cells in S. mansoni infected DEREG mice upon asthma induction. In A) the efficacy of Treg depletion was controlled by analyzing the percentage of cells in peripheral blood by flow cytometry (see Figure 1E). In brief, prior to depletion (d50, upper panel) and 3 days after DT injections (d53, lower panel) the percentage of CD4+egfp+ T cells was observed in S. mansoni infected (left) and naive DEREG mice (right). B) Upon asthma induction, the percentage of Treg was observed again in peripheral blood (d74). Representative dot plot on the left depicts the levels of CD4+egfp+ T cells in a Inf/OVADT mouse and the right image those observed in a OVADT mouse. C) Bars represent the mean + SEM of CD4+egfp+ T cells on d74 recovered from 4–5 mice per group. Percentages were calculated by flow cytometry. Asterisks show statistical differences (Student's t test) between the groups indicated by the brackets (**p<0.01). (TIF) [file pntd.0002379.s004.tif]
